# Variability of linezolid concentrations after standard dosing in critically ill patients: a prospective observational study

**DOI:** 10.1186/cc13984

**Published:** 2014-07-10

**Authors:** Michael Zoller, Barbara Maier, Cyrill Hornuss, Christina Neugebauer, Gundula Döbbeler, Dorothea Nagel, Lesca Miriam Holdt, Mathias Bruegel, Thomas Weig, Béatrice Grabein, Lorenz Frey, Daniel Teupser, Michael Vogeser, Johannes Zander

**Affiliations:** 1 Department of Anesthesiology, Hospital of the Ludwig-Maximilians-University of Munich, Munich, Germany; 2 Institute of Laboratory Medicine, Hospital of the Ludwig-Maximilians-University of Munich, Marchioninistrasse 15, Munich 81377, Germany; 3 Department of Clinical Microbiology and Hospital Hygiene, Hospital of the Ludwig-Maximilians-University of Munich, Munich, Germany

## Abstract

**Introduction:**

Severe infections in intensive care patients show high morbidity and mortality rates. Linezolid is an antimicrobial drug frequently used in critically ill patients. Recent data indicates that there might be high variability of linezolid serum concentrations in intensive care patients receiving standard doses. This study was aimed to evaluate whether standard dosing of linezolid leads to therapeutic serum concentrations in critically ill patients.

**Methods:**

In this prospective observational study, 30 critically ill adult patients with suspected infections received standard dosing of 600 mg linezolid intravenously twice a day. Over 4 days, multiple serum samples were obtained from each patient, in order to determine the linezolid concentrations by liquid chromatography tandem mass spectrometry.

**Results:**

A high variability of serum linezolid concentrations was observed (range of area under the linezolid concentration time curve over 24 hours (AUC_24_) 50.1 to 453.9 mg/L, median 143.3 mg*h/L; range of trough concentrations (C_min_) < 0.13 to 14.49 mg/L, median 2.06 mg/L). Furthermore, potentially subtherapeutic linezolid concentrations over 24 hours and at single time points (defined according to the literature as AUC_24_ < 200 mg*h/L and C_min_ < 2 mg/L) were observed for 63% and 50% of the patients, respectively. Finally, potentially toxic levels (defined as AUC_24_ > 400 mg*h/L and C_min_ > 10 mg/L) were observed for 7 of the patients.

**Conclusions:**

A high variability of linezolid serum concentrations with a substantial percentage of potentially subtherapeutic levels was observed in intensive care patients. The findings suggest that therapeutic drug monitoring of linezolid might be helpful for adequate dosing of linezolid in critically ill patients.

**Trial registration:**

Clinicaltrials.gov NCT01793012. Registered 24 January 2013.

## Introduction

Severe infections in ICU patients remain a major challenge in modern medicine. The prevalence of severe infections such as sepsis or septic shock in ICU patients ranges from 20 to 80% with high mortality rates of 20 to 50% [1-5]. Consequently, there is a substantial need for optimizing antimicrobial therapy. Key elements for the treatment of infections include an adequate antimicrobial therapy with an early initiation and with sufficiently high drug concentration levels [6-10]. Furthermore, sufficiently high drug concentrations are required to limit the development of antimicrobial resistance [11].

About 50% of bloodstream infections in critically ill patients are caused by Gram-positive bacteria [12,13]. A major part of these Gram-positive infections are represented by multidrug-resistant strains (for example, methicillin-resistant *Staphylococcus aureus* (MRSA) and vancomycin-resistant enterococci (VRE)), which are particularly frequent in ICUs [12-17]. Linezolid has good *in vitro* and *in vivo* activity against these organisms and is an important antibiotic for the treatment of infections in critically ill patients [9,16,18-22]. The volume of distribution in adults of this hydrophilic antibiotic approximates to the total body water content of 40 to 60 L [23]. The plasma elimination half-life is mostly reported to be between 3.1 and 4.9 h with a clearance rate between 6.4 and 14.8 L/h [23]. Linezolid is metabolized by liver enzymes to two major inactive metabolites, an aminoethoxyacetic acid and a hydroxyethyl glycine, which are excreted predominantly - together with the parent substance - in urine [24,25]. Because of its intrinsic chemico-physical and pharmacokinetic characteristics, it is assumed that adequate serum linezolid concentrations will be achieved most of the time when using the recommended dose of 600 mg every 12 hours and that therapeutic drug monitoring (TDM) might not be necessary [26,27]. This assumption is based on reports showing adequate linezolid concentrations in healthy volunteers or non-critically ill patients [27-29]. According to the manufacturer, no dose adjustment of linezolid is necessary in the case of renal or liver impairment. Consequently, expert panels recommend standardized doses of 600 mg linezolid twice a day also for patients with severe infections such as sepsis or septic shock [30]. However, 10 to 30% of critically ill patients receiving linezolid have treatment failure despite isolation of Gram-positive organisms sensitive to linezolid [16,31,32]. Of these, 15 to 30% of patients suffer furthermore from adverse effects such as elevated liver enzymes, gastrointestinal disturbances or hematological toxicity [32-34]. The rate of therapy failure and adverse effects may be in part explained by a high variability of linezolid serum concentrations in critically ill patients.

Though there are have been few studies evaluating blood levels and pharmacokinetic parameters of linezolid in critically ill patients [26,35-41], it is still very difficult for physicians to decide if therapeutic levels are reached after standard dosing with linezolid when treating critically ill patients. The studies to date have found variable results with regard to linezolid blood levels. A substantial number demonstrated that inadequate levels occur [36,37,39,41] whereas others concluded that standard doses are mostly sufficient [35,38]. Low numbers of study patients, the lack of use of compartment models, and the retrospective design of most studies leave inconclusive information about this topic within the existing literature. Moreover, there are only preliminary data for linezolid blood levels in specific subgroups of ICU patients, such as critically ill patients on continuous renal replacement therapy (CRRT), those on extracorporeal lung assist (ECLA) and patients who have undergone organ transplantation [41,42]. Indeed, most of these studies excluded particular patient groups, therefore, do not represent the full spectrum of different patients in ICUs. We therefore designed a prospective observational study to analyze the variability of linezolid serum concentrations in relation to preliminary target concentration ranges in a heterogeneous group of critically ill patients with suspected infections. The primary aim of the study was to evaluate whether linezolid serum concentrations in different critically ill patients were within the defined therapeutic range.

## Materials and methods

### Patients

The study population originated from medical-surgical critically ill patients hospitalized in two ICUs within the Department of Anesthesiology, University Hospital of Munich between March and November 2013. Patients were eligible for inclusion if they had a severe infection (confirmed or suspected by clinical assessment) and were treated with linezolid intravenously by short-duration infusions according to the clinic guidelines, and in accordance with the German Paul-Ehrlich-Society and the guidelines of the Infectious Disease Society of America [30,43]. Patients were only excluded if they were under the age of 18 years, if their planned hospitalization was less than 4 days, or if the first linezolid administration was given more than 48 h before study enrollment. Written informed consent was obtained from all patients or their legal representatives.

### Study design

The monocentric, prospective observational study was performed at the University Hospital of Munich. The study protocol (ClinicalTrials.gov, NCT01793012) was approved by the Institutional Review Board of the Medical Faculty of the Ludwig-Maximilians-University (registration number 428-12) and carried out according to the principles of the Declaration of Helsinki. Enrolled patients (n = 30) received 600 mg linezolid twice a day by short-duration infusion (15 to 60 minutes). Day 1 of the study was defined as that day on which the first linezolid trough level (C_min_) was determined (see Additional file 1). This was directly before the second or third linezolid administration in all patients except patients 2 and 27, for whom the study start was directly before the fifth and fourth linezolid administration, respectively. Serum samples from the arterial line for antibiotic determination were collected at multiple time points before (C_min_), during, and after the two linezolid administrations on day 1; and before, during, and after one of the two linezolid administrations on days 2, 3 and 4 (in total 26 to 43 samples per patient). The exact time of blood sampling was recorded by the medical staff. Samples were immediately sent to the Institute of Laboratory Medicine, University Hospital of Munich, centrifuged (3,000 g, 10 minutes) and aliquoted into 2-ml polypropylene tubes (Eppendorf, Hamburg, Germany). Serum aliquots were stored within one hour after blood sampling at -80°C.

### Determination of clinical and laboratory parameters

Clinical patient data and diagnosis in the ICU were recorded. Sepsis was defined according to the Society of Critical Care Medicine/European Society of Intensive Care Medicine (SCCM/ESICM) Consensus Conference Committee [10]. The severity of the patient’s clinical condition was characterized using the acute physiology and chronic health evaluation (APACHE) II score. To assess renal function, creatinine concentrations in both serum and 24-h urine samples were determined using an enzymatic photometric test on an automated chemistry analyzer (Model AU5822: Beckmann Coulter, Brea, CA, USA). Creatinine clearance (CL_crea_) was calculated using the formula:

$${\mathrm{CL}}_{\mathrm{crea}}=\left(C_{\mathrm{urine}}*V_{\mathrm{urine}}\right):\left(C_{\mathrm{serum}}*\mathrm{time}\right),$$

where C_urine_ is the creatinine concentration in urine, V_urine_ is the urine volume, and C_serum_ is the serum creatinine concentration.

### Determination of linezolid concentrations

Serum linezolid concentrations were determined using a previously described liquid chromatography tandem mass spectrometry (LC-MS/MS) method [44]. Briefly, sample preparation was based on protein precipitation and on-line solid phase extraction with two-dimensional liquid chromatography and column switching. Three-fold deuterated linezolid was used as the internal standard. Control samples were used from both a commercial provider and from in-house production. Validation revealed good analytical performance showing inaccuracy <6% and imprecision <7.3% (coefficient of variation) for all quality control samples. The lower limit of quantification was 0.13 mg/L. The method was found to be robust over the course of the study.

### Pharmacokinetic analysis

We analyzed linezolid plasma concentrations with a compartmental pharmacokinetic model based on nonlinear mixed-effects modeling. For model estimation we used the NONMEM 7.2® program (Icon Development Solutions, Hanover, MD, USA) with the FOCE-I estimation algorithm. The aim of the pharmacokinetic analysis was to determine individual concentration time courses. We assumed that the population parameters were log-normally distributed. The individual post-hoc concentration predictions obtained from NONMEM were used to predict the time course of linezolid plasma concentrations and to calculate the area under the concentration time curve over 24 h (AUC_24_)-values. Model selection was based on the NONMEM objective function, goodness-of-fit plots, and median absolute performance errors as described by Varvel *et al*. [45]. For graphical analysis we used PLTTools 5.0 PLTsoft, San Francisco, CA USA [46]. Linezolid plasma concentrations were calculated for each patient based on individual pharmacokinetic parameters in 10-minute steps.

### Assessment of target concentration ranges

The thresholds for potential therapeutic efficacy were defined as C_min_ >2 mg/L and/or AUC_24_ > 200 mg*h/L. The rationale behind these two thresholds was the findings of Rayner *et al*., showing a higher therapeutic success in seriously ill patients when linezolid exceeds the minimum inhibitory concentration (MIC) over the entire dosing interval or when AUC_24_/MIC-values are higher than 80 to 120 [31]. We defined the MIC as the concentration that inhibits the growth of 90% of important relevant infectious pathogens (MIC_90_) (particularly *S. aureus* and Enterococcus species) [47,48] and therefore, we set the threshold of potential therapeutic efficacy of C_min_ at 2 mg/L. As in Rayner *et al*., we set the AUC_24_/MIC_90_ value at 100, corresponding to a threshold for potential therapeutic efficacy of AUC_24_-values of 200 mg*h/L. The threshold for potential therapeutic toxicity was defined as trough levels >10 mg/L or AUC_24_ values >400 mg*h/L according to the literature [23,26,34].

### Statistics

The AUC_24_ was calculated by means of the trapezoidal rule using concentration values as predicted by the pharmacokinetic model (individual post-hoc concentration time course). Patients were divided into three groups in relation to the defined target concentration ranges based on their C_min_- and AUC_24_-values. Non-continuous parameters were expressed as percentages and numbers, and compared by means of the Chi-square test. Continuous parameters were expressed as median values and ranges, and compared by the Jonckheere-Terpstra test. A *P*-value below 0.05 was considered statistically significant. All calculations were performed using SAS (version 9.3, SAS Institute Inc., Cary, NC, USA).

## Results

Twenty male and ten female patients with a median age of 57 years (range, 28 to 84 years) and a median body mass index (BMI) of 25.5 kg/m^2^ (range, 16 to 35 kg/m^2^) were included. The most frequent causes of sepsis were pneumonia and peritonitis (Table 1). Ten patients were lung-transplant, and five were liver-transplant recipients. Patients had high variability in APACHE II scores (range 9 to 37, median 27.5). Of the 25 patients who were not on CRRT, 5 had a reduced creatinine clearance of <50 ml/minute. Five patients were being treated with CRRT and seven patients were treated with ECLA. Detailed parameters of the corresponding CRRT and ECLA systems are shown in Additional files 2 and 3.

**Table 1:** Characteristics of the study population

**Patient number**	**APACHE II score**	**Mean creatinine clearance (ml/minute)**^**a**^	**CRRT (+/-)**	**ECLA (+/-)**	**Organ transplantation**^**b**^	**Clinical condition**
1	20	89	-	+	Lung	Septic pneumonia
2	12	129	-	-	-	ARDS
3	20	21	-	-	-	Septic peritonitis
4	28	114	-	-	-	Septic pleural empyema
5	23	97	-	+	-	ARDS
6	33	72	-	+	-	Septic pneumonia
7	9	117	-	-	Lung	Septic pneumonia
8	28	119	-	-	Lung	Septic pneumonia
9	28	-	+	-	Liver	Septic peritonitis
10	29	94	-	-	-	Septic pneumonia
11	33	35	-	-	Lung	Septic pneumonia
12	31	102	-	+	-	Septic pneumonia
13	14	127	-	-	-	Septic pneumonia
14	14	69	-	-	Lung	Septic pneumonia
15	32	42	-	-	-	Septic endocarditis
16	19	76	-	+	-	Septic pneumonia
17	35	-	+	-	Liver	Septic peritonitis
18	21	33	-	-	Lung	Septic pneumonia
19	27	55	-	-	Lung	Septic pneumonia
20	17	64	-	-	Lung	Septic pleural empyema
21	30	68	-	-	-	Septic pneumonia
22	23	-	+	-	Liver	Septic peritonitis
23	14	85	-	-	Liver	Septic peritonitis
24	24	-	+	-	Liver	Septic peritonitis
25	37	74	-	-	Lung	Septic pneumonia
26	25	83	-	+	Lung	Septic pneumonia
27	28	-	+	-	-	Septic peritonitis
28	34	163	-	+	-	Septic pneumonia
29	32	126	-	-	-	ARDS
30	29	37	-	-	-	Septic peritonitis
Median	27.5	83				
(range)	(9 to 37)	(21 to 163)				

^a^Mean value of the 4 study days; ^b^within the last 28 days. APACHE II, acute physiology and chronic health evaluation II score; CRRT, continuous renal replacement therapy; ECLA, extracorporeal lung assist; ARDS, acute respiratory distress syndrome.

Linezolid plasma concentrations were best described by a two-compartment model with an individual (post-hoc) median prediction error of 1% and a median absolute prediction error of 13%. The parameter estimates (standard error) of the population model were: volume of distribution of the first compartment = 19.3 (3.9) L, volume of distribution of the second compartment = 26.4 (3.8) L, elimination clearance = 8.3 (0.9) L/h and inter-compartmental clearance = 56.0 (19.3) L/h.

**Figure 1:**
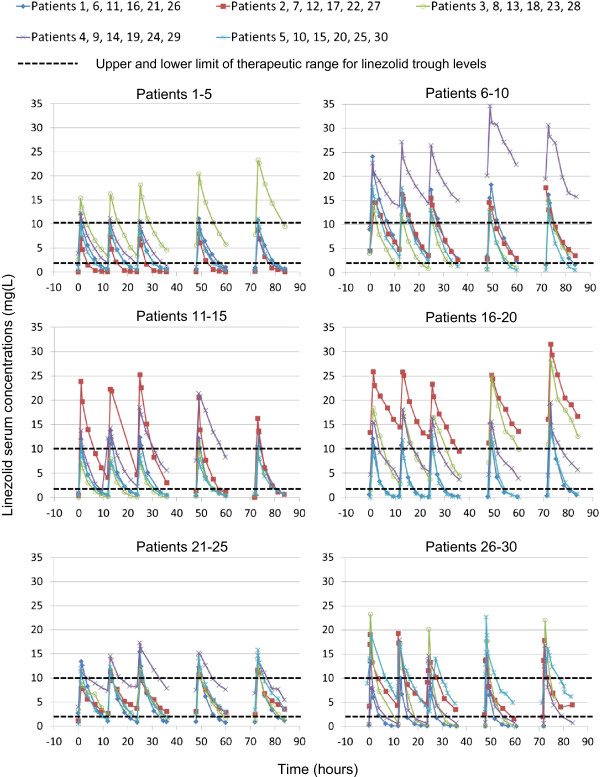
**Serum concentration profiles of the 30 study patients.** Serum concentrations over the course of 4 days, as measured by liquid chromatography tandem mass spectrometry are shown.

Figure 1 shows the concentration time curves of serum linezolid for each patient. A high inter-patient variability was observed. The high inter-patient variability could be quantified when AUC_24_-values ranged from 50.1 to 453.9 mg*h/L (median 143.3 mg*h/L) (Table 2). The high inter-patient variability was also observed for single C_min_-values (range >100-fold, from <0.13 to 14.49 mg/L, median 2.06 mg/L) (Table 2). To obtain further information about the usefulness of c_min_-values for TDM, C_min_ values were correlated with corresponding AUC_24_ values giving an *r*^2^ value of 0.79 (Additional file 4).

**Table 2:** Linezolid pharmacokinetic parameters following intravenous administration of 600 mg twice daily in critically ill ICU patients

**Patient number**	**AUC**_**24 **_(mg*h/L)^**a**^	**C**_**min **_**(mg/L)**^**b**^
1	99.1	0.67
2	50.1	<0.13^c^
3	258.2	3.35
4	118.3	1.89
5	82.3	0.46
6	213.7	3.15
7	206.3	3.59
8	138.9	1.17
9	453.9	13.75
10	165.3	3.26
11	105.3	0.43
12	224.7	4.13
13	67.6	0.14
14	266.9	2.31
15	80.8	0.70
16	101.7	0.18
17	442.0	14.49
18	325.6	2.75
19	217.8	3.48
20	94.8	<0.13
21	122.0	1.03
22	144.6	3.70
23	141.8	2.22
24	250.8	8.18
25	157.1	1.45
26	50.8	<0.13
27	165.1	4.22
28	106.2	0.17
29	86.5	0.45
30	244.1	5.86
Median	143.3	2.06
Range	50.1 to 453.9	<0.13 to 14.49

^a^As determined by the NONMEM system, values from the start of the third administration of linezolid over 24 h; ^b^as analyzed by liquid chromatography tandem mass spectrometry, values obtained directly before the fourth administration of linezolid if not indicated otherwise;

^c^value obtained directly before the fifth administration of linezolid. AUC_24_, concentration time curve over 24 h; C_min_, linezolid trough level.

In addition to the inter-patient variability, high intra-patient variability of C_min_ values was also observed. Nine of thirty patients had maximum C_min_ values, more than 5-fold higher than the minimum C_min_ values (range of maximum/minimum C_min_ values 1 to 36) (Figure 1; see also Additional file 5). The C_min_ of most patients did not change in a consistent pattern over the 4 days of the study; only in patients 3, 14, 18 and 25 did we observe an increase, and in patient 4 a decrease of C_min_ values over time (Figure 1).

Optimal pharmacodynamic exposure over 24 h with AUC_24_ values between 200 and 400 mg*h/L and at a single time point with C_min_ values between 2 and 10 mg/L was observed in 30% and 43% of the patients, respectively (Table 3, Figure 1). Regarding these AUC_24_ and C_min_ values, 63% and 50% of the study patients had linezolid concentrations below the lower limit of the corresponding target concentration range, respectively, and 7% had linezolid concentrations above the target concentration range. Moreover, only 17% of the patients continuously attained optimal C_min_ values between 2 and 10 mg/L over 4 days (see Additional file 5).

**Table 3:** Distribution of patients in relation to the target range of linezolid

	**Number (percentage) of linezolid patients**					
**Patient groups, number of patients**	**AUC**_**24**_, mg*h/L^**a**^			**C**_**min**_**, mg/L**^**b**^		
	**<200**	**200 to 400**	**>400**	**<2**	**2 to 10**	**>10**
Total patients, n =30	19 (63)	9 (30)	2 (7)	15 (50)	13 (43)	2 (7)
Male patients, n = 20^c^	13 (65)	5 (25)	2 (10)	11 (55)	7 (35)	2 (10)
Female patients, n = 10	6 (60)	4 (40)		4 (40)	6 (60)	
On CRRT, n = 5^d^	2 (40)	1 (20)	2 (40)		3 (60)	2 (40)
Not on CRRT, n = 25	17 (68)	8 (32)		15 (60)	10 (40)	
On ECLA, n = 7^c^	5 (71)	2 (29)		5 (71)	2 (29)	
Not on ECLA, n = 23	14 (61)	7 (30)	2 (9)	10 (43)	11 (48)	2 (9)
After liver transplantation, n = 5^f^	2 (40)	1 (20)	2 (40)		3 (60)	2 (40)
After lung transplantation, n = 10^c^	6 (60)	4 (40)		6 (60)	4 (40)	
No transplantation, n = 15	11 (73)	4 (27)		9 (60)	6 (40)	

^a^As determined by the NONMEM system, values from the start of the third administration of linezolid; ^b^as determined by liquid chromatography tandem mass spectrometry, values obtained directly before the fourth administration of linezolid; ^c^no significantly different values in comparison to the corresponding patient group; ^d^significantly higher values in patients on CRRT than in those not on CRRT (*P* <0.01); ^f^significantly higher values in patients with liver transplantation than in non-liver-transplant patients (*P* <0.05). AUC_24_, concentration time curve over 24 h; C_min_, linezolid trough level; CRRT, continuous renal replacement therapy; ECLA, extracorporeal lung assist.

Patients on CRRT had significantly higher C_min_ values and AUC_24_ values than patients without CRRT (*P* = 0.005 for AUC_24_ and *P* = 0.001 for C_min_) (Table 3). Similarly, patients who had undergone liver transplantation had significantly higher AUC_24_ and C_min_ values than in non- (liver and lung) transplant patients (*P* = 0.036 for AUC_24_ and *P* = 0.012 for C_min_). Other characteristics such as gender, lung transplantation and therapy with ECLA did not have any significant influence on AUC_24_ or C_min_ values. Variability of linezolid levels was high in patient groups on CRRT and ECLA, and in the liver and lung transplantation groups, with each group showing a substantial proportion (≥40%) outside the target concentration range. The distributions of continuous parameters in relation to linezolid target ranges are shown in Table 4. A trend towards higher linezolid serum levels was observed in patients with reduced creatinine clearance, although these changes were not significant (*P* = 0.102 for C_min_ and *P* = 0.051 for AUC_24_) (Table 4, Additional file 6). Other continuous parameters such as age, BMI, and APACHE-II score did not have any significant influence on AUC_24_ or C_min_ values.

**Table 4:** Distributions of continuous parameters in relation to linezolid target ranges

	**Median and range of patients**					
	**AUC**_**24 **_(mg*h/L)^**a**^			**C**_**min **_**(mg/L)**^**b**^		
**Patient groups**	**<200**	**200 to 400**	**>400**	**<2**	**2 to 10**	**>10**
Mean creatinine clearance (mL/minute)^c^						
median	89	62		89	70	
range	35 to 163	21 to 117		35 to 163	21 to 117	
(number of patients)	(17)	(8)		(15)	(10)	
APACHE II score						
median	28	24	32	28	24	32
range	12 to 37	9 to 33	28 to 35	12 to 37	9 to 33	28 to 35
(number of patients)	(19)	(9)	(2)	(15)	(13)	(2)
Age (years)						
median	53	59	56	57	57	56
range	28 to 77	34 to 84	50 to 61	28 to 77	29 to 84	50 to 61
(number of patients)	(19)	(9)	(2)	(15)	(13)	(2)
Body mass index (kg/m^2^)						
median	24	19	21	22	23	21
range	13 to 32	17 to 26	19 to 23	13 to 32	17 to 28	19 to 23
(number of patients)	(19)	(9)	(2)	(15)	(13)	(2)

^a^As determined by the NONMEM system, values from the start of the third administration of linezolid; ^b^as determined by liquid chromatography tandem mass spectrometry, values obtained directly before the fourth administration of linezolid; ^c^mean value of the 4 study days. AUC_24_, concentration time curve over 24 h; C_min_, linezolid trough level; APACHE II, acute physiology and chronic health evaluation II.

## Discussion

This study shows that the recommended standard dosing of linezolid leads to subtherapeutic linezolid plasma concentrations in about every second critically ill patient. Furthermore, a high variability of linezolid levels was observed in the study population with a majority (57 to 70%) detected outside the target ranges. Although there were insufficient levels in the majority of patients, inappropriate high levels occurred in a small number of patients. Finally, this variability of linezolid levels was not only observed between the different patients, but also within individual patients over the course of 4 days.

Our findings are in line with other studies also observing very low AUC_24_ or C_min_ values with the majority being insufficient [37,39,41]. In contrast to some other studies [37,39], we used two different approaches to define the lower threshold of the target range. First, we used AUC_24_/MIC in concordance with other studies [26,35,36,38,40,41], which was shown to be the best parameter to predict efficacy [31]. When we used this approach 63% of our study patients had insufficient linezolid serum levels. Second, we used C_min_ values as a further lower threshold of the target concentration range, as time above the MIC over the entire dosing interval also correlates with efficacy [31]. This value was set at 2 mg/L in accordance with MIC_90_ values of relevant causative pathogens [47,48] as had been done in other studies [26,37,39]. Different efficacy thresholds might be used in environments where MIC_90_ values of relevant pathogens differ from 2 mg/L. Indeed, linezolid serum concentrations during infection should reach sufficient levels for most causative pathogens to ensure efficacy. This might be particularly important, as the identity of most causative pathogens is unknown in the early course of severe infection.

Furthermore, we showed that there is a high variability of linezolid AUC_24_ and C_min_ values with C_min_ values differing more than 100-fold between the different study patients and more than 30-fold within individual patients. This is in line with some recent studies also describing high variability of C_min_ values differing more than 50-fold between different patients [26,34,41]. In fact, the majority of linezolid concentrations in our study was outside the defined linezolid target concentration ranges, supporting the concept of TDM. We set the upper threshold of the target concentration range for C_min_ values at 10 mg/L. This was done in accordance with other studies, because higher concentrations have been shown to be associated with drug-related toxicity [23,26,34], whereas Pea *at al*. showed that in long-term treatment with linezolid an upper threshold of the target concentration range of 7 mg/L should be favored [49]. Indeed, in the study of Pea *et al*., all patients with C_min_ values >10 mg/L of linezolid had substantial platelet reduction (>30%) during long-term linezolid treatment, whereas no patient had these adverse effects with C_min_ values <4 mg/L. Despite the rather high upper threshold used in our study, 7% of the patients had linezolid concentrations above the target range. The fact that for critically ill patients, the two parameters AUC_24_/MIC >80 to 120 and time above MIC over the entire dosing interval strongly correlated with treatment efficacy [31], and that elevated linezolid concentrations correlated with adverse effects [23,34], show that both AUC_24_ and C_min_ values correlate with efficacy and toxicity. This strongly supports the concept of linezolid target concentration ranges in terms of TDM. The good linear relationship between C_min_ and AUC_24_ values described by Pea *et al*. [26] was confirmed in our study (*r*^2^ = 0.79). C_min_ might therefore be a useful parameter for TDM of linezolid in clinical practice. As a high variation of C_min_ values within individual patients was observed in this study, we would recommend repetitive determinations of linezolid C_min_ values during infection treatment.

Finally, the linezolid serum concentrations in different critically ill patients, such as those on CRRT or ECLA, and patients who had undergone liver or lung transplantation, were evaluated. Only a few studies have evaluated the pharmacokinetics of linezolid in critically ill patients on CRRT [50-53], thereby using different CRRT systems such as continuous venovenous hemodiafiltration (CVVHDF) and continuous venovenous hemofiltration (CVVH). Linezolid concentrations were partly subtherapeutic and partly within the potential therapeutic range, however, a comparison of linezolid levels in patients not on CRRT was not performed. In our study, linezolid concentrations were tested in patients with CVVHDF and CVVHD. In comparison to the other study patients, significantly higher linezolid levels were observed in patients on CRRT. However, it should be noted that four of the five patients on CRRT were liver transplant recipients and that higher levels of linezolid have been reported in patients after liver transplantation [41]. About 50% of the parent substance linezolid is metabolized by liver enzymes to two major inactive metabolites and are excreted predominantly - together with linezolid - in urine [25]. Higher levels of linezolid in patients after liver transplantations might therefore be due to alterations in the activity of liver enzymes after ischemia/reperfusion [41]. Higher numbers of patients on CRRT and those after liver transplantations with simultaneous evaluation of the liver function, as well as of linezolid and its inactive metabolites in urine will be necessary to definitely understand the impact of CRRT and liver transplantations on linezolid concentrations in critically ill patients. Furthermore, we evaluated linezolid levels in patients after lung transplantation and patients on ECLA, and no significant differences (in the setting used in our study) in comparison to the whole study population were observed. Admittedly, the majority of the linezolid levels were also insufficient in these patients. This is in line with the reported low linezolid levels in three critically ill patients on extracorporeal membrane oxygenation-systems (specific ECLA system) and three critically ill patients after lung transplantation [42,50], which are the only available data on these patients.

The results of this study suggest that the limited availability of linezolid quantification methods in clinical laboratories might pose a serious problem for the antimicrobial therapy of ICU patients. The reason for the limited availability of such methods is in particular the lack of cost-efficient commercially available linezolid quantification tests. Only a few laboratories use custom-made linezolid quantification methods such as high performance liquid chromatography with UV-detection (HPLC-UV) or LC-MS/MS. Development and routine use of these custom-made methods require a high level of human resources, professional specialization and high-technology equipment, which can often only be provided by large or specialized laboratories. The availability of commercial quantification tests (for example, based on HPLC-UV or immunoassay methods) of a therapeutic substance applied in life-threatening conditions might therefore be of particular impact.

The present study considered a number of aspects which have only partially been covered in previous work: (a) we investigated numerous critically ill patients from the whole intensive care spectrum, including patients who had undergone lung and liver transplantation and during CRRT and ECLA; (b) we analyzed a large number of linezolid plasma samples. This allowed description of individual concentration time courses by pharmacokinetic modeling with a small median prediction error of 1% and a small median absolute prediction error of 13%; (c) finally, this study used a highly accurate method for linezolid quantification [44]. All other studies measuring linezolid concentrations in critically ill patients used HPLC-UV [26,35-41], which may be prone to interference, especially in critically ill patients with extended co-medication. In contrast, we used an LC-MS/MS method, thereby, for the first time, using isotope dilution internal standardization. As target analytes and internal standards are very similar in their physico-chemical properties, variances of individual samples impacting the ionization are compensated almost completely, realizing the highest attainable level of reliability [54]. Furthermore, the use of control samples from both a commercial provider and from in-house production additionally ensures the accuracy of the method. Indeed, there were only minimal undulations in the concentration-time curves of linezolid in individual patients (Figure 1), showing that the study-protocol, including blood sampling and analytical method, was accurately performed.

The high variability of linezolid levels found in our study, with a substantial proportion at insufficient low levels, might contribute to the observed high mortality rate and severity of infection in ICU patients. Furthermore, high variability of linezolid levels may also lead to the development of resistance and drug-related toxicity. As early and effective antimicrobial therapy has a substantial effect on bacterial eradication and patient survival [6,31], optimal individual dosing of antibiotics is of particular importance. Given the fact that the most common cause of death in the ICU in medically advanced nations is severe infection [55] and because of worldwide intentions to reduce morbidity and mortality from sepsis [10], we believe that there is great importance in optimizing individual antimicrobial dosing with the aid of TDM.

## Conclusions

We found high variability in linezolid serum concentrations with mostly insufficient low levels in critically ill patients. We therefore suggest general TDM of linezolid in critically ill patients during linezolid therapy. However, future studies will have to investigate whether application of TDM can definitely improve linezolid-dosing protocols and infection-related patient outcome.

## Key messages

• High variability of linezolid serum concentrations after standard linezolid dosing in 30 different critically ill patients with suspected infections were observed.

• We observed potentially subtherapeutic levels in the majority of different patients.

• Potentially toxic levels were observed in a minority of different patients.

• Our data suggests that therapeutic drug monitoring might be helpful for adequate dosing of linezolid in critically ill patients.

## Abbreviations

APACHE: acute physiology and chronic health evaluation; AUC_24_: concentration time curve over 24 h; BMI: body mass index; C_min_: linezolid trough level; CRRT: continuous renal replacement therapy; CVVH: continuous venovenous hemofiltration; CVVHD: Continuous venovenous hemodialysis; CVVHDF: continuous venovenous hemodiafiltration; ECLA: extracorporeal lung assist; HPLC-UV: high performance liquid chromatography with UV-detection; LC-MS/MS: liquid chromatography tandem mass spectrometry; MIC: minimal inhibitory concentration; MRSA: methicillin-resistant *Staphylococcus aureus*; SCCM/ESICM: Society of Critical Care Medicine/European Society of Intensive Care Medicine; TDM: therapeutic drug monitoring; VRE: vancomycin-resistant enterococci.

## Competing interests

The authors declare that they have no competing interests.

## Authors’ contributions

MZ and JZ designed the study and wrote the manuscript; BM measured antibiotic concentrations by LC-MS/MS; CN and GD were responsible for acquisition of data; DN and CH performed the statistical analyses; LMH, MB, TW, BG, LF, DT and MV made substantial contributions to the conception and design of the study including interpretation of results. All authors critically revised the manuscript for important intellectual content and approved the final manuscript. All authors meet key authorship requirements and agree to be accountable for all aspects of the work in ensuring that questions related to the accuracy or integrity of any part of the work are appropriately investigated and resolved.

## Supplementary Material

Additional file 1 **Figure showing study protocol of blood sampling for linezolid determination. **^a^Two to three linezolid infusions before study start with the exception of patients 2 and 27, for whom the study start was directly before the fifth and fourth linezolid administration, respectively: ^b^26 to 43 samples per patient. Click here for file

Additional file 2 Table showing parameters of the continuous renal replacement therapy systems used for each patient in this study. Click here for file

Additional file 3 Table showing parameters of the extracorporeal lung-assist systems used for each patient in this study. Click here for file

Additional file 4 **Figure showing correlation of linezolid trough level (C**_**min**_**) values and concentration time curve over 24 h (AUC**_**24**_**) values of linezolid.** Shown are values for AUC_24_ as determined by the NONMEM system from the beginning of the third administration of linezolid, and C_min_ as determined by liquid chromatography tandem mass spectrometry (LC-MS/MS) directly before the fourth administration of linezolid. Click here for file

Additional file 5 **Figure demonstrating the high variability (inter-patient and intrapatient) of linezolid trough levels (C**_**min**_**) over the course of the study for each patient.** Click here for file

Additional file 6 **Table showing concentration time curve over 24 h (AUC**_**24**_**) and linezolid trough level (C**_**min**_**) values of patients from the lowest and highest quartile of covariates.** Click here for file
