# Supplementary figures and images for: Variability of linezolid concentrations after standard dosing in critically ill patients: a prospective observational study
Source: Crit Care. 2014 Jul 10;18(4):R148. doi: 10.1186/cc13984 (PMC4227093; doi:10.1186/cc13984)

## Slide 1
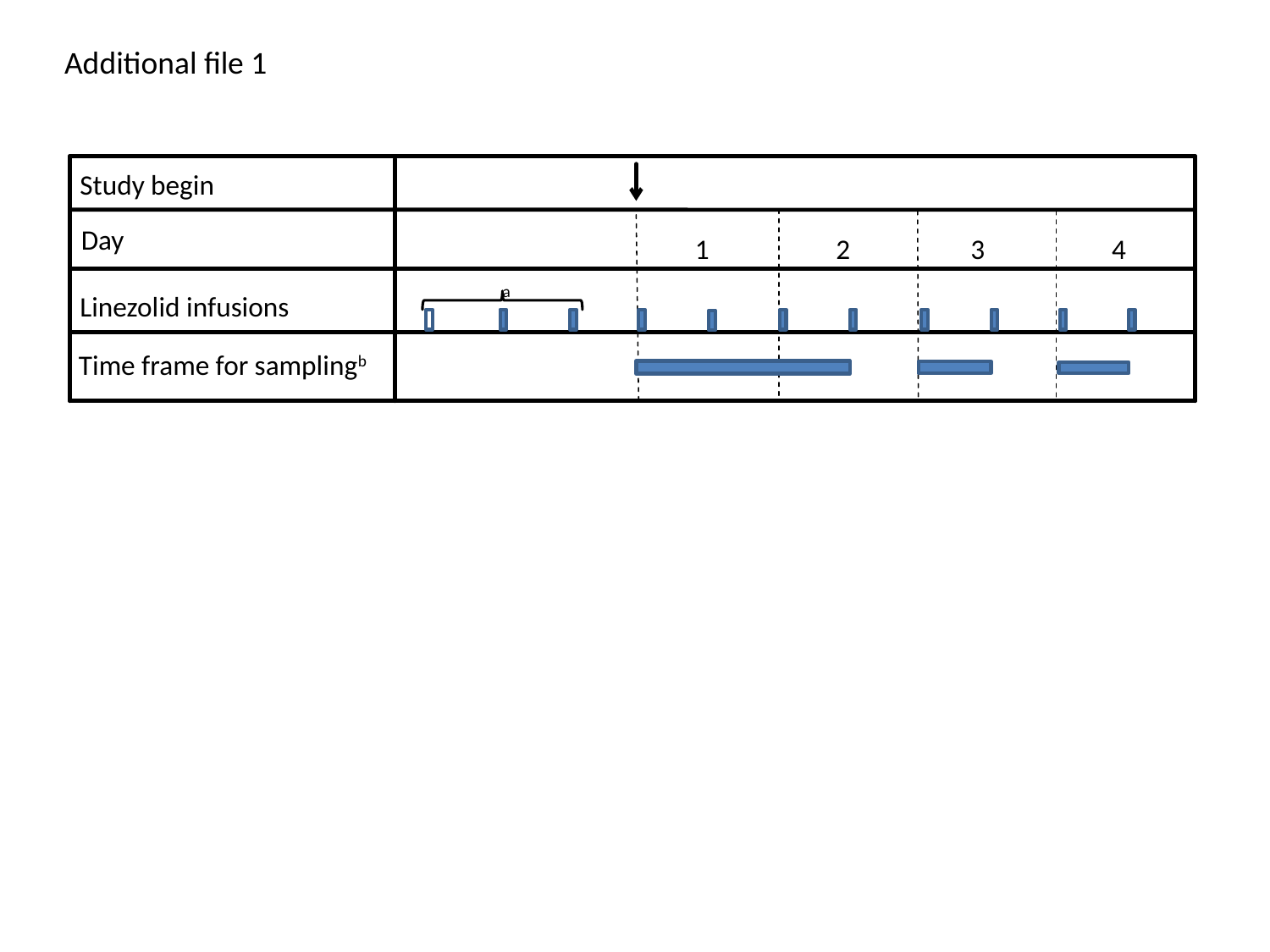

Additional file 1
Study begin
Day
1 2 3 4
a
Linezolid infusions
Time frame for samplingb

Supplement: Additional file 1 — Figure showing study protocol of blood sampling for linezolid determination. aTwo to three linezolid infusions before study start with the exception of patients 2 and 27, for whom the study start was directly before the fifth and fourth linezolid administration, respectively: b26 to 43 samples per patient. [file cc13984-S1.pptx]

## Slide 1
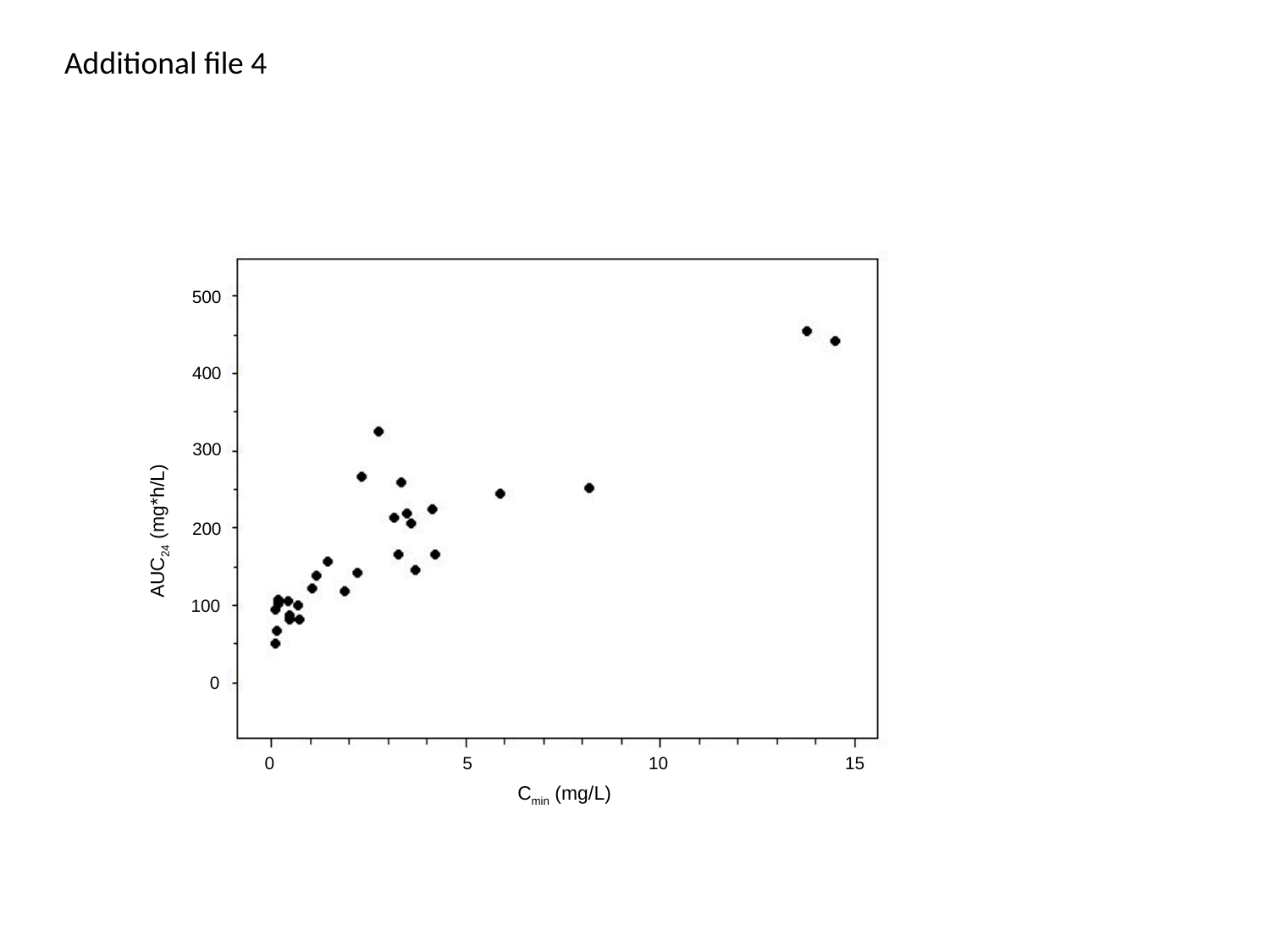

Additional file 4
500
400
AUC24 (mg*h/L)
300
200
100
0
0
5
10
15
Cmin (mg/L)

Supplement: Additional file 4 — Figure showing correlation of linezolid trough level (Cmin) values and concentration time curve over 24 h (AUC24) values of linezolid. Shown are values for AUC24 as determined by the NONMEM system from the beginning of the third administration of linezolid, and Cmin as determined by liquid chromatography tandem mass spectrometry (LC-MS/MS) directly before the fourth administration of linezolid. [file cc13984-S4.pptx]
