# Supplementary material for: Variability of linezolid concentrations after standard dosing in critically ill patients: a prospective observational study
Source: Crit Care. 2014 Jul 10;18(4):R148. doi: 10.1186/cc13984 (PMC4227093; doi:10.1186/cc13984)
Supplement: Additional file 2 — Table showing parameters of the continuous renal replacement therapy systems used for each patient in this study. [file cc13984-S2.docx]

Additional file 2: Parameters of the continuous renal replacement therapy systems used in this study

| **Patient number** | **Mode of  CRRT^a^** | **Dialysate rate (ml/h)** | **Filtration rate (ml/h)** | **Blood flow (ml/min)** | **Fluid removal (ml/h)** | **Duration^b^ of CRRT use (h)** |
| --- | --- | --- | --- | --- | --- | --- |
| 9 | CVVHD^c^ | 2000 | - | 100-120 | 50-100 | 76 |
| 17 | CVVHDF^d^ | 1500 | 1500 | 100-130 | 0-200 | 48 |
| 22 | CVVHD | 2000 | - | 80-120 | 100-200 | 78 |
| 24 | CVVHD | 1000-2000 | - | 150 | 80 | 96 |
| 27 | CVVHDF | 1200 | 1000-2000 | 150 | 50-250 | 85 |

^a^, continuous renal replacement therapy; ^b^, within the 4 days of the study; ^c^, continuous venovenous hemodialysis, used with Ultraflux® AV-1000S-filters (Fresenius, Bad Homburg, Germany); ^d^, continuous venovenous hemodiafiltration, used with Ultraflux® AV-600S-filters (Fresenius).
