# Supplementary material for: Variability of linezolid concentrations after standard dosing in critically ill patients: a prospective observational study
Source: Crit Care. 2014 Jul 10;18(4):R148. doi: 10.1186/cc13984 (PMC4227093; doi:10.1186/cc13984)
Supplement: Additional file 3 — Table showing parameters of the extracorporeal lung-assist systems used for each patient in this study. [file cc13984-S3.docx]

Additional file 3: Parameters of the extracorporeal lung assist systems used in this study

| **Patient number** | **Mode of ECLA^a^** | **Blood flow (l/min)** | **Gas flow (l/min)** | **Duration^b^ of ECLA use (h)** |
| --- | --- | --- | --- | --- |
| 1 | VV-ECMO^c^ | 4.0-4.8 | 6.0-7.0 | 73 |
| 5 | VV-ECMO | 2.0 | 2.0 | 36 |
| 6 | ECCO_2_R^d^ | 0.37-0.45 | 3.0-6.5 | 96 |
| 12 | VV-ECMO | 2.8-3.5 | 3.0-4.0 | 96 |
| 16 | VV-ECMO | 3.0-4.0 | 6.0-8.0 | 96 |
| 26 | VV-ECMO | 1.5-2.0 | 3.0-3.5 | 41 |
| 28 | pECLA^e^ | 0.8-1.1 | 4.0-10.0 | 96 |

^a^, extracorporeal lung assist; ^b^, within the 4 days of the study; ^c^, venovenous extracorporeal membrane oxygenation, used with Maquet Rotaflow centrifugal pump and BE-PLS 2050 circuit (Maquet, Rastatt, Germany); ^d^, minimally invasive extracorporeal CO2 removal, used with Hemolung RAS® Technology (ALung Technologies, Pittsburgh, PA, USA); ^e^, pumpless extracorporeal lung assist, used with iLA membrane ventilator Novalung® IL 1000-01 (Novalung, Heilbronn, Germany).
