# Supplementary material for: Variability of linezolid concentrations after standard dosing in critically ill patients: a prospective observational study
Source: Crit Care. 2014 Jul 10;18(4):R148. doi: 10.1186/cc13984 (PMC4227093; doi:10.1186/cc13984)
Supplement: Additional file 5 — Figure demonstrating the high variability (inter-patient and intrapatient) of linezolid trough levels (C min ) over the course of the study for each patient. [file cc13984-S5.pptx]

## Slide 1
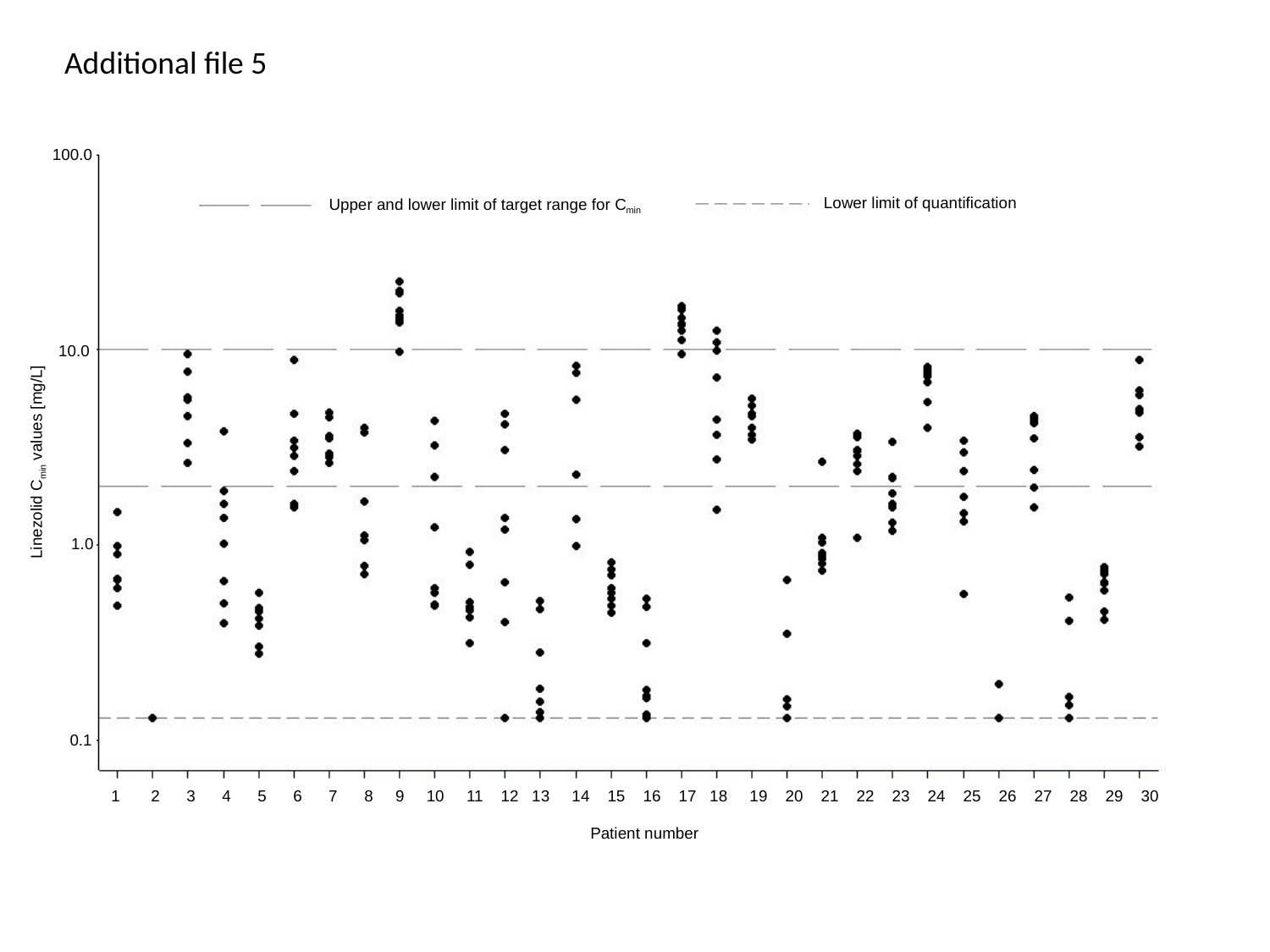

Additional file 5
100.0
Lower limit of quantification
Upper and lower limit of target range for Cmin
10.0
Linezolid Cmin values [mg/L]
1.0
0.1
 1 2 3 4 5 6 7 8 9 10 11 12 13 14 15 16 17 18 19 20 21 22 23 24 25 26 27 28 29 30
Patient number
