# Supplementary material for: Variability of linezolid concentrations after standard dosing in critically ill patients: a prospective observational study
Source: Crit Care. 2014 Jul 10;18(4):R148. doi: 10.1186/cc13984 (PMC4227093; doi:10.1186/cc13984)
Supplement: Additional file 6 — Table showing concentration time curve over 24 h (AUC 24 ) and linezolid trough level (C min ) values of patients from the lowest and highest quartile of covariates. [file cc13984-S6.docx]

Additional File 6: AUC_24_- and C_min_-values of patients from the lowest and highest quartile of covariates

|  | **Covariates** | | | | | | | | | | |
| --- | --- | --- | --- | --- | --- | --- | --- | --- | --- | --- | --- |
|  | **Creatinine clearance^a^** | |  | **APACHE-II score** | |  | **Age** | |  | **BMI** | |
| **Linezolid level   parameter** | **quartile** | |  | **quartile** | |  | **quartile** | |  | **quartile** | |
|  | **lowest** | **highest** |  | **lowest** | **highest** |  | **lowest** | **highest** |  | **lowest** | **highest** |
| AUC-values^b^ (mg*h/L) |  |  |  |  |  |  |  |  |  |  |  |
| median | 231 | 96 |  | 102 | 106 |  | 106 | 214 |  | 106 | 118 |
| range | 81-326 | 50-206 |  | 50-267 | 81-442 |  | 95-251 | 81-267 |  | 95-326 | 50-244 |
|  |  |  |  |  |  |  |  |  |  |  |  |
| C_min_^c^ (mg/L) |  |  |  |  |  |  |  |  |  |  |  |
| median | 3.1 | 0.31 |  | 0.18 | 0.70 |  | 0.43 | 2.3 |  | 0.43 | 1.9 |
| range | 0.4-5.9 | <0.13-3.6 |  | <0.13-3.6 | 0.17-14.5 |  | <0.13-8.2 | 0.45-5.9 |  | <0.13-3.6 | <0.13-5.9 |

^a^, mean value of the 4 study days

^b^, as determined by NONMEM, values from begin of the third administration of linezolid;
^c^, as determined by LC-MS/MS, values directly before the fourth administration of linezolid;
